# Holiness Is Health

**Published:** 1871-11

**Authors:** 


### HOLINESS IS HEALTH.

That is, a holy life involves a temperate and industrious life, and temperance and industry bring health and happiness the world over.

What is true as to the whole is true as to part; in proportion as men are holy in the above sense, they are healthy and happy. Of " wisdom," symbolizing religion, it is said, " Length of days is in her right hand, and in her left riches and honor." " Thou shalt come to thy grave in a full age, like as a shock of corn cometh in his season." Of the irreligious it is said, " They shall not live out half their days."

There is reason to believe that people who go regularly to " meeting " on Sundays, live longer, on an average, than those who do not go to church at all, even though they be not members of the church; because men cannot hear the preaching of the gospel regularly, without its having a restraining influence over their conduct, whether they are willing to acknowledge it or not. A man may hear a sermon, and when he gets home he may not be able to remember distinctly a single idea; at the same time, he heard many things, and as some of these passed across his mind, they left impressions; they left an influence which will hold a rein on the conduct; for Divinity has said of the preached word, "It shall not return unto me void; but it shall accomplish that which I please." The bare fact of a man's dressing up to go to church, does him good; every man is elevated by being dressed well, presupposing that it is preceded by a good wash all over, these being palpable advances in the direction of health. The little Indian who asked for a "young" shirt, meaning a small one, in contradistinction of a large one which had been given to his father, and not knowing how to ask for a little one for himself, felt as big as a king, as soon as he put it on.

Then there is the repose and rest from worldly toil, and more or less of worldly care, when we enter the sanctuary. We may hear an important practical truth and not think of it again; but in a week, a month, a year afterwards, it starts up before us with remarkable freshness sometimes, at the very moment its use is called for. Now let us put these things to the test of figures and facts. An intelligent gentleman, living in a flourishing town in New England, kept a record of all the deaths in fifteen years; some of the tabulated results are as follows:—

In fifteen years, no church member under twenty years of age died.

Of 202 who died over twenty years of age, 90 were church members and 112 were not. Five of the 90 church members lived to the age of 90 years, only 2 of the 112 non-church members, reached that age; 18 church members lived to be over 80, and only 11 non-professors.

The "Congregational Quarterly" makes out the following very suggestive statement. Out of —

| | | |
|---|---|---|
| 100 professors of religion | . . . | 71 lived over 90 |
| 100 non-professors of " | . . . | 29 only " 90 |
| 100 professors " " | . . . | 62 lived over 80 |
| 100 non-professors " " | . . . | 38 " " 80 |
| 100 professors " " | . . . | 64 " " 70 |
| 100 non-professors " " | . . . | 36 " " 70 |
| 100 professors " " | . . . | 56 " " 60 |
| 100 non-professors " " | . . . | 44 " " 60 |

Of 100 professors under 60, thirty-four were church members, 66 were not.

Out of 100 who were under 50, 33 were church members and 67 were not.

Out of 100 deaths under 40 years of age, 27 were church members, 73 were not.

Out of 100 who died under 30 years of age, 23 were church members, 77 were not.

It is very much to be desired that clergymen, or other intelligent persons of villages, would keep similar accounts, and report them to their own denominational paper, for the purpose of ascertaining on a large scale if it be true that professors of religion, even although some of them are notoriously irreligious, have a longer life, on an average, than non-professors. If so, the promotion of hygiene is a necessary result of a religious life, verifying the Bible statement, that "Godliness hath the promise of the life that now is, and of that which is to come."

